# Pathogenic characteristics of central nervous system infections in AIDS individuals: a retrospective cohort study based on immune status

**DOI:** 10.3389/fneur.2026.1743821

**Published:** 2026-03-11

**Authors:** Qing-Nan Xu, Chen-Fan Liu, Xiao-Yan Wang, Jun-Ming Gao

**Affiliations:** Department of Infectious Diseases II, Shandong Public Health Clinical Center, Jinan, Shandong, China

**Keywords:** AIDS, central nervous system infections, clinical outcomes, immune status, pathogen

## Abstract

**Background:**

Human immunodeficiency virus (HIV) infection frequently leads to central nervous system (CNS) complications, especially as immunity declines. While antiretroviral therapy (ART) has transformed HIV into a manageable condition, in clinical practice, a significant subset of individuals still present with advanced immunosuppression (CD4 < 200 cells/μL) due to delayed diagnosis, treatment failure, or poor adherence, leading to AIDS-defining conditions. This study investigated how immune status affects CNS infection characteristics and outcomes specifically within this immunocompromised spectrum of AIDS individuals, aiming to provide refined evidence for risk stratification and early intervention in late-presenting or treatment-experienced individuals.

**Methods:**

We retrospectively analyzed 213 individuals with AIDS who presented with CNS symptoms and confirmed CNS infections. They were grouped by CD4 count: 101 with ≥200 cells/μL (Moderate to High Immune, HI) and 112 with <200 cells/μL (Low Immune, LI). We compared symptoms, cerebrospinal fluid (CSF) findings, imaging results, detected pathogens, treatment outcomes, and quality of life between groups.

**Results:**

Individuals with low CD4 counts showed significantly more severe symptoms like altered consciousness (50.00% vs. 31.68%, *p* = 0.007) and seizures (25.00% vs. 12.87%, *p* = 0.025). Their CSF tests revealed higher white blood cell counts (30.72 ± 8.50 vs. 18.32 ± 4.41 cells/μL, *p* < 0.001), higher protein levels (85.21 ± 32.14 vs. 62.36 ± 20.53 mg/dL, *p* < 0.001), lower glucose levels (41.35 ± 13.40 vs. 50.12 ± 10.24 mg/dL, *p* < 0.001), and higher pathogen detection rates (53.57% vs. 26.73%, *p* < 0.001). The CD4 < 200 group also had significantly lower CD4/CD8 ratios (*p* < 0.001). Brain magnetic resonance imaging (MRI) also revealed a higher prevalence of abnormalities in the LI group, including parenchymal lesions, ventriculomegaly, meningeal enhancement, and focal lesions (*p* < 0.05). Opportunistic infections, particularly *Cryptococcus neoformans* (49.11% vs. 22.77%, *p* < 0.001), *Mycobacterium tuberculosis* (MTB) (18.75% vs. 7.92%, *p* = 0.021), and Cytomegalovirus (CMV) (15.18% vs. 5.94%, *p* = 0.030), were much more common in the LI group. Individuals in the MHI group achieved complete remission more often (66.34% vs. 51.79%, *p* = 0.031) and had less disease progression (9.90% vs. 21.42%, *p* = 0.022). Their quality of life scores after treatment for the CNS infection were also significantly better across physical, psychological, social, and overall health domains (*p* < 0.05). Multivariate analysis confirmed that a higher CD4 count strongly protected against poor outcomes (OR = 0.321 per 100 cells/μL increase, *p* < 0.001).

**Conclusion:**

This study quantifies the severe impact of advanced immunosuppression (CD4 < 200 cells/μL) on CNS infection presentation, short-term treatment response (for CNS infections), and quality of life at discharge. Preventing immune decline below this threshold is critical.

## 1 Introduction

Acquired Immunodeficiency Syndrome (AIDS), caused by the Human Immunodeficiency Virus (HIV), is associated with substantial global morbidity and mortality, affecting approximately 39 million people worldwide and accounting for an estimated 0.63 million deaths annually (1, 2). While antiretroviral therapy (ART) has transformed HIV into a manageable condition, individuals remain at risk of severe complications, including infections of the central nervous system (CNS) (3). The CNS is recognized as a reservoir for HIV and is vulnerable to both direct HIV-related injury and opportunistic infections (4).

The immune system, particularly CD4 + T-cells, is closely associated with immune defense against pathogens (5). However, the relationship between immune status and both susceptibility to and severity of CNS infections has not been fully characterized (6). While previous studies have shown that low CD4 counts increase the risk of CNS infections, few studies have examined clinical and laboratory characteristics of CNS infections across defined CD4-based immune states, incorporating modern diagnostic and treatment contexts (7).

CNS infections in HIV individuals are often caused by opportunistic pathogens such as *Cryptococcus neoformans*, *Mycobacterium tuberculosis* (MTB), and Cytomegalovirus (CMV) (8). CNS involvement by these pathogens is more commonly observed in the context of impaired immune function and may result in clinical manifestations such as meningitis, encephalitis, or focal lesions (9). CD4 + T-cells are closely associated with pathogen-specific immune responses against these pathogens (10). For example, *Cryptococcus neoformans* requires Th1-type immunity mediated by CD4 + T-cells to control fungal growth, while MTB relies on cell-mediated immunity to contain granulomas (11). Similarly, CMV reactivation is closely tied to CD8 + T-cell function, which is itself modulated by CD4 + T-cell help (12). Declining CD4 counts not only reduce the ability to combat infections but also impair pathogen clearance, which can be accompanied by increased inflammation and tissue damage (13).

Despite advances in ART, CNS infections remain a significant cause of morbidity and mortality in people living with HIV (14). This study aimed to examine the association between immune status and the prevalence, severity, and outcomes of CNS infections in HIV individuals. By analyzing clinical data, CSF profiles, and neuroimaging findings, we sought to identify key immune-related predictors of poor prognosis and inform clinical risk stratification and management. Understanding these relationships could help prioritize interventions for individuals at highest risk, ultimately improving survival and quality of life.

## 2 Materials and methods

### 2.1 Study design and ethics statement

This study was a retrospective cohort study that analyzed the clinical data of consecutively eligible HIV individuals treated at the Department of Infectious Diseases II, Shandong Public Health Clinical Center from July 2020 to June 2025. Based on CD4 cell count levels, individuals were divided into two groups: 101 individuals with CD4 cell counts ≥ 200 cells/μL were classified as the moderate to high immune status group (MHI group), and 112 individuals with CD4 cell counts < 200 cells/μL were classified as the low immune status group (LI group). The threshold of 200 cells/μL was selected based on established AIDS-defining criteria and clinical guidelines indicating increased risk of opportunistic infections below this level (15). All participants received standard-of-care antimicrobial therapy for their specific CNS infection.

This study was approved by the Institutional Review Board (IRB) of Shandong Public Health Clinical Center (approval no. GWLCZXEC-SOP-K-2025-140), and was conducted in accordance to the tenets of the Declaration of Helsinki. Given that the study was retrospective in design, using only anonymized medical records without involving additional interventions for individuals, and posed no potential harm or impact on individuals, the ethics committee waived the requirement for informed consent. The personal privacy of all participants was strictly protected to ensure the confidentiality and security of the data.

### 2.2 Inclusion and exclusion criteria

Inclusion Criteria: Individuals met the diagnostic criteria for AIDS (16), with a documented positive HIV test result; were aged between 18 and 65 years; had complete clinical data; and during the study period, exhibited CNS-related symptoms (such as headache, altered consciousness, seizures, etc.), with CNS lesions confirmed by microbiological methods (e.g., cerebrospinal fluid analysis, blood cultures, or tissue biopsy) or standardized clinical diagnostic criteria (e.g., clinical presentation combined with imaging studies).

Exclusion Criteria: Individuals meeting any of the following conditions were excluded from the study: incomplete medical records, unable to provide complete medical history and laboratory test results; CNS symptoms primarily caused by non-infectious factors (such as tumors-including primary CNS lymphoma, which was differentiated from infection by imaging characteristics, CSF cytology, and/or biopsy when indicated, metabolic diseases, drug toxicity, or other non-infectious inflammation); concurrent severe diseases (such as non-CNS malignancies, autoimmune diseases, etc.); participation in other studies or treatment protocols that could affect CNS infections, unless these interventions were well-documented and did not significantly interfere with the data analysis of this study; pregnant or breastfeeding women.

### 2.3 Data collection

All data were extracted from electronic medical records (EMR). Collected data included demographics, clinical symptoms, laboratory results (including CD4+/CD8 + counts, HIV viral load, cerebrospinal fluid analysis), neuroimaging findings, identified pathogens, and clinical outcomes. Information on antiretroviral therapy (ART) was also extracted, specifically focusing on whether the participant was receiving ART at the time of admission for the CNS infection (yes/no). Detailed ART history, including specific regimens and precise duration of therapy, was variably documented in the EMR and was not included as a formal analyzed variable in this retrospective study, as the key immunological parameter of interest was the CD4 count at presentation. Notably, there were no missing data for any of these variables, ensuring robust and complete analyses. The absence of missing data enhances the validity and reliability of our statistical results.

### 2.4 Measurement of immunological and virological parameters

(1) CD4+/CD8 + T-cell counts: quantified using flow cytometry (Agilent NovoCyte^®^; Agilent Technologies, United States).
(2) HIV Viral Load: Quantified using reverse transcription polymerase chain reaction (RT-PCR) (COBAS^®^ TaqMan^®^ HIV-1 Test v2.0; Roche Diagnostics, Switzerland; detection limit: 20 copies/mL).

### 2.5 Cerebrospinal fluid (CSF) analysis

(1) Cell count: manual counting using a Neubauer hemocytometer (Paul Marienfeld GmbH, Germany) within 1 h of collection.
(2) Biochemical analysis: the protein levels were quantified using Pandy’s reagent via the turbidimetric method, while glucose levels were measured using Fujifilm reagents via the hexokinase method. Both analyses were performed on a Cobas^®^ c501 analyzer (Roche Diagnostics, Switzerland), ensuring accurate and reliable assessment of these biochemical parameters in our study samples.
(3) CNS pathogen detection (all individuals underwent a standardized CSF pathogen testing panel):

*Cryptococcus neoformans*: CSF underwent India ink staining (Zhuhai Baso Biotechnology Co., Ltd., China), cryptococcal antigen lateral flow assay (IMMY CrAg^®^ LFA; Danaher Biotech Co., Ltd., China), and culture on Sabouraud dextrose agar (Oxoid^™^; Thermo Fisher Scientific, United States).
*Mycobacterium tuberculosis* (MTB): CSF tested using acid-fast staining (BD BBL^™^ TB Ziehl-Neelsen Kit; Becton Dickinson, United States), Löwenstein-Jensen culture (BD BBL^™^; Becton Dickinson, United States), and GeneXpert MTB/RIF Ultra (Cepheid, United States).
John Cunningham Virus (JCV)/Cytomegalovirus (CMV): Identified by Multiplex PCR (FastTrack Diagnostics^®^ CNS Infection Panel, Siemens Healthineers, Germany).

### 2.6 Neuroimaging protocol

Brain MRI (3.0 T GE Signa^™^ Architect; GE Healthcare, United States) was performed with and without contrast enhancement. Images were independently reviewed by two blinded neuroradiologists, with discrepancies resolved by consensus. Findings documented and analyzed included: Brain parenchymal lesions (e.g., masses, ring-enhancing lesions, diffuse signal abnormalities); Ventriculomegaly; Meningeal enhancement (leptomeningeal or pachymeningeal); Focal lesions (e.g., abscesses, tuberculomas, infarcts).

### 2.7 Clinical outcomes of CNS infections (assessed at discharge)

The treatment response for the diagnosed CNS opportunistic infection was assessed at discharge and categorized as follows:

(1) Complete Remission (CR): Resolution of presenting CNS symptoms and significant improvement or resolution of objective findings (imaging/CSF abnormalities).
(2) Partial Remission (PR): Marked improvement in symptoms and objective findings but not meeting criteria for CR.
(3) Disease Progression (DP): Worsening of symptoms, development of new neurological deficits, or deterioration in imaging/CSF findings despite treatment (17).

### 2.8 Quality of life (QoL) assessment (assessed at discharge)

Health-related Quality of Life (QoL) was assessed using the validated Mandarin-adapted Medical Outcomes Study HIV Health Survey (MOS-HIV), which evaluates four domains: Physical Function, Psychological Function, Social Function, and Overall Health. Responses were scored on Likert scales and converted to a 0–100 scale, with higher scores indicating better QoL. The survey demonstrated good reliability (Cronbach’s *α* > 0.80) in this cohort (18, 19).

### 2.9 Statistical analysis

Data were analyzed using SPSS 29.0 statistical software (SPSS Inc., Chicago, IL, United States). Normality was assessed using the Shapiro–Wilk test. For continuous data that followed a normal distribution, results are presented as mean ± standard deviation (X ± s). Differences between the MHI group and the LI group were compared using t-tests. Categorical variables are reported as frequencies and percentages [*n* (%)] and were analyzed using chi-square (χ^2^) tests or Fisher’s exact test where appropriate. Viral load values were log_10_-transformed for statistical analysis but reported in standard units (copies/mL) throughout. Multivariable logistic regression analysis was performed to identify independent predictors of poor prognosis (defined as disease progression or partial remission). Variables included in the model were selected based on clinical relevance and univariate associations (*p* < 0.10), and included CD4 count (per 100 cells/μL), HIV viral load (log₁₀ copies/mL), presence of severe CNS symptoms (altered consciousness or seizures), pathogen type (*Cryptococcus neoformans* vs. others), and ART usage at admission. Collinearity was assessed using variance inflation factors (all <2.5). The results are presented as odds ratios (OR) with corresponding 95% confidence intervals (CI) and *p* values. No adjustment for multiple comparisons was made for exploratory analyses. All tests were two-tailed, with a *p* value less than 0.05 considered statistically significant.

## 3 Results

### 3.1 Demographic characteristics

In Table 1, we compared the demographic characteristics between the MHI group (*n* = 101) and the LI group (*n* = 112). Our analysis showed no significant differences in age, gender distribution, ethnicity, BMI, education level, residence type, smoking history, or drinking history between the two groups (all *p* > 0.05). These results indicate that the two groups were similar in terms of demographic characteristics.

**Table 1:** Comparison of demographic characteristics between two groups.

Parameter	MHI group (*n* = 101)	LI group (*n* = 112)	t/χ^2^	*P*
Age (years)	26.50 ± 3.23	25.85 ± 3.10	1.503	0.134
Gender (male/female) [*n*(%)]	84 (83.17%)	94 (83.93%)	0.022	0.881
Ethnicity (Han/other) [*n*(%)]	93 (92.08%)	104 (92.86%)	0.046	0.83
BMI (kg/m^2^)	23.10 ± 3.10	22.85 ± 3.30	0.555	0.58
Education level (high school or below/junior college or above) [*n*(%)]	56 (55.45%)	63 (56.25%)	0.014	0.906
Residence (urban/rural) [*n*(%)]	61 (60.40%)	68 (60.71%)	0.002	0.962
Smoking history [*n*(%)]	30 (29.70%)	34 (30.36%)	0.011	0.917
Drinking history [*n*(%)]	24 (23.76%)	27 (24.11%)	0.003	0.953

BMI: Body Mass Index.

### 3.2 Clinical characteristics

As presented in Table 2, significant differences were observed in several key clinical characteristics between the two groups, consistent with the grouping criteria. Specifically, the MHI group had significantly higher CD4 + T counts (*p* < 0.001), lower CD8 + T counts (*p* < 0.001), higher CD4/CD8 ratios (*p* < 0.001), and lower HIV viral loads (*p* < 0.001) compared to the LI group. There were no significant differences noted in the rates of co-infections, ART usage rate at admission, or history of previous CNS infections (all *p* > 0.05).

**Table 2:** Comparison of clinical characteristics between two groups.

Parameter	MHI group (*n* = 101)	LI group (*n* = 112)	t/χ^2^	*P*
CD4 + T count (cells/μL)	252.15 ± 65.12	38.50 ± 10.21	32.613	<0.001
CD8 + T count (cells/μL)	890.23 ± 220.06	1200.09 ± 350.11	7.810	<0.001
CD4/CD8 ratio	0.35 ± 0.12	0.08 ± 0.03	21.901	<0.001
HIV viral load (log₁₀ copies/mL)	3.81 ± 1.20	5.22 ± 1.51	7.583	<0.001
Co-infections [*n*(%)]	27 (26.73%)	37 (33.04%)	1.004	0.316
ART usage rate at admission [*n*(%)]	31 (30.69%)	23 (20.54%)	2.895	0.089
History of previous CNS infections [*n*(%)]	16 (15.84%)	24 (21.43%)	1.087	0.297

CD4 + T: Cluster of Differentiation 4-positive T Lymphocytes; CD8 + T: Cluster of Differentiation 8-positive T Lymphocytes; ART: Antiretroviral Therapy; CNS: Central Nervous System.

### 3.3 Distribution of presenting symptoms

**Figure 1:**
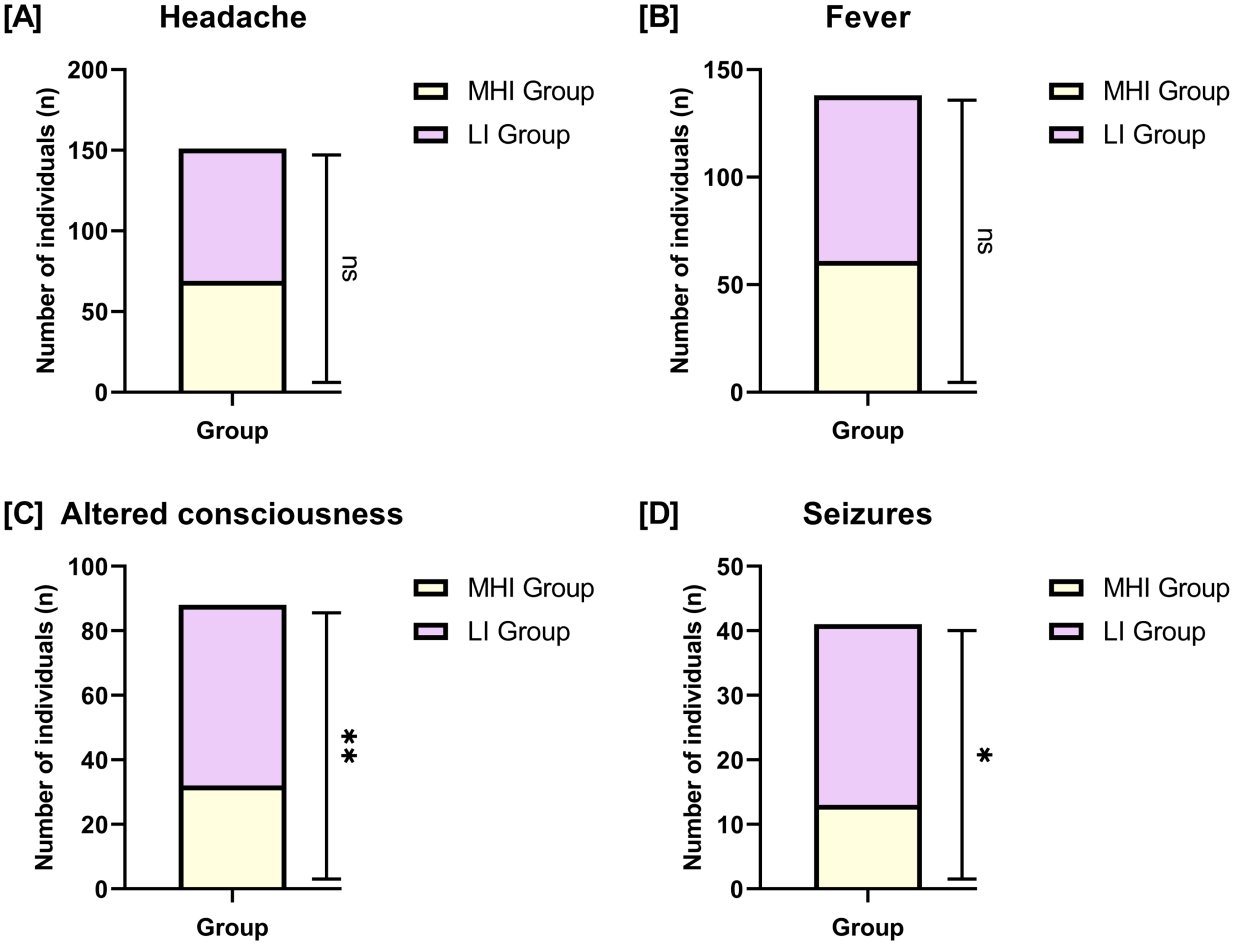
Comparison of presenting symptoms between two groups. **(A)** Headache; **(B)** fever; **(C)** altered consciousness; **(D)** seizures. Bars represent the number of individuals with each symptom in the MHI group and LI group. Sample sizes: MHI group, *n* = 101; LI group, *n* = 112. NS: No significant difference; *: *p* < 0.05; **: *p* < 0.01.

In Figure 1, we compared the prevalence of key presenting symptoms between the MHI group and the LI group. There were no significant differences in the rates of headache (*p* = 0.432) or fever (*p* = 0.202) between the two groups. However, significant differences were observed in the rates of altered consciousness (MHI group: 31.68% vs. LI group: 50.00%, *p* = 0.007) and seizures (MHI group: 12.87% vs. LI group: 25.00%, *p* = 0.025).

### 3.4 Imaging and cerebrospinal fluid (CSF) analysis results

In Table 3, we examined the differences in neuroimaging findings between the MHI group and the LI group. The prevalence of brain parenchyma lesions was significantly higher in the LI group compared to the MHI group (*p* = 0.006). Similarly, ventriculomegaly was more common in the LI group (*p* = 0.017). Meningeal enhancement also exhibited significant variation (*p* = 0.008). Additionally, focal lesions were more frequently observed in the LI group than in the MHI group (*p* = 0.047).

**Table 3:** Comparison of imaging results between two groups.

Parameter	MHI group (*n* = 101)	LI group (*n* = 112)	χ^2^	*P*
Brain parenchyma lesions [*n*(%)]	19 (18.81%)	40 (35.71%)	7.576	0.006
Ventriculomegaly [*n*(%)]	13 (12.87%)	29 (25.89%)	5.689	0.017
Meningeal enhancement [*n*(%)]	35 (34.65%)	59 (52.68%)	6.998	0.008
Focal lesions [*n*(%)]	18 (17.82%)	33 (29.46%)	3.953	0.047

In Table 4, we compared CSF analysis results between the MHI group and the LI group. The white blood cell count in CSF was notably higher in the LI group compared to the MHI group (*p* < 0.001). Similarly, protein levels were elevated in the LI group relative to the MHI group (*p* < 0.001). Conversely, glucose levels were lower in the LI group than in the MHI group (*p* < 0.001). Additionally, the positivity rate for pathogen detection was significantly greater in the LI group compared to the MHI group (*p* < 0.001).

**Table 4:** Comparison of cerebrospinal fluid analysis results between two groups.

Parameter	MHI group (*n* = 101)	LI group (*n* = 112)	t/χ^2^	*P*
White blood cell count (cells/μL)	18.32 ± 4.41	30.72 ± 8.50	13.543	<0.001
Protein levels (mg/dL)	62.36 ± 20.53	85.21 ± 32.14	6.243	<0.001
Glucose levels (mg/dL)	50.12 ± 10.24	41.35 ± 13.40	5.594	<0.001
Pathogen detection positivity rate	27 (26.73%)	60 (53.57%)	15.833	<0.001

### 3.5 Pathogen detection rate in central nervous system (CNS) infection

**Figure 2:**
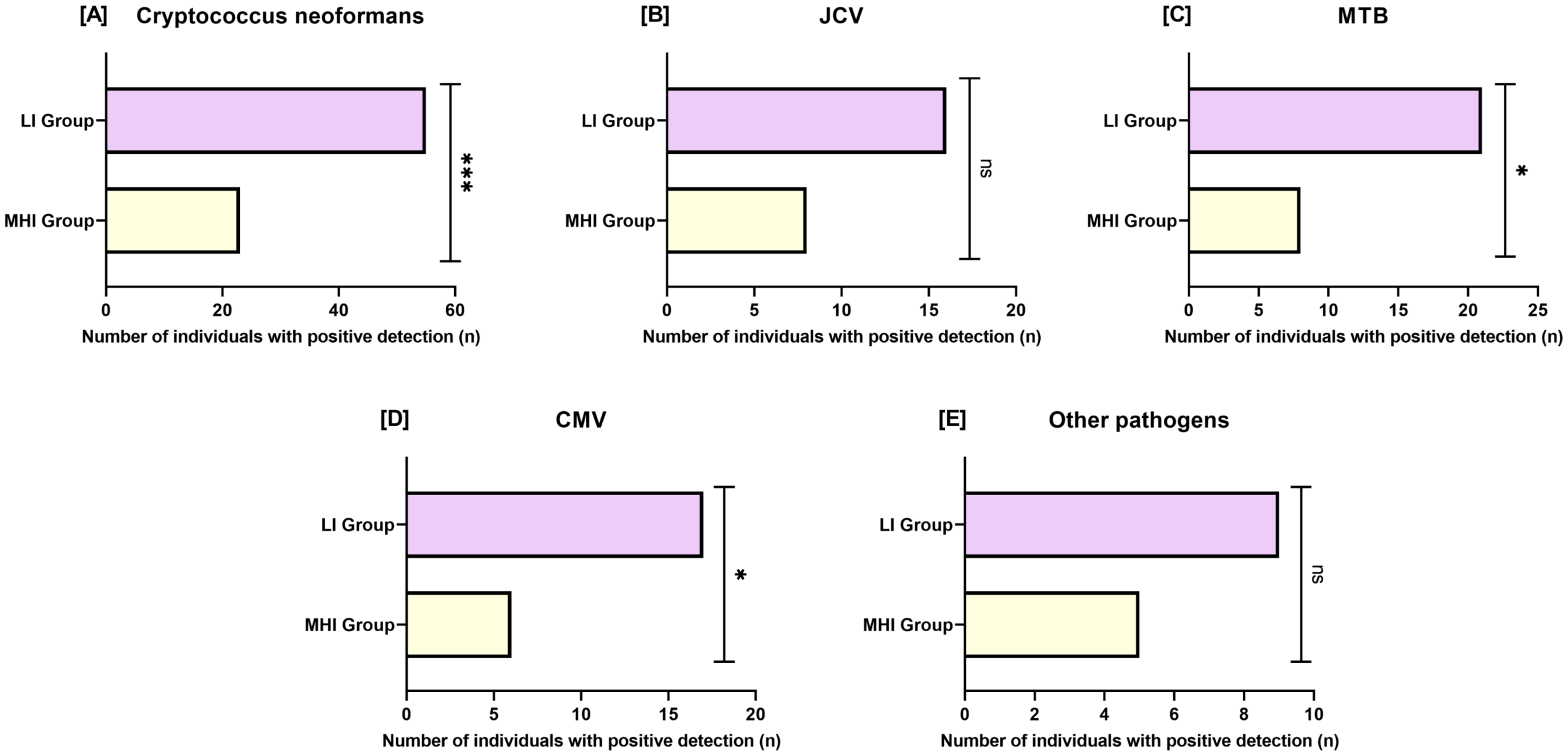
Comparison of pathogen detection rate between two groups. **(A)**: *Cryptococcus neoformans*; **(B)**: JCV; **(C)**: MTB; **(D)**: CMV; **(E)**: Other pathogens. JCV: John Cunningham virus; MTB: *Mycobacterium tuberculosis*; CMV: Cytomegalovirus. Bars represent the number of individuals with positive pathogen detection. Sample sizes: MHI group, *n* = 101; LI group, *n* = 112. Ns: No significant difference; *: *p* < 0.05; ***: *p* < 0.001.

In Figure 2, we compared the detection rates of various pathogens between the MHI group and the LI group. Higher detection frequencies of *Cryptococcus neoformans* (MHI group: 22.77% vs. LI group: 49.11%, *p* < 0.001), MTB (MHI group: 7.92% vs. LI group: 18.75%, *p* = 0.021), and CMV (MHI group: 5.94% vs. LI group: 15.18%, *p* = 0.030) were observed in the LI group. In contrast, no significant differences were found in the detection rates of JCV (*p* = 0.142) or other pathogens (*p* = 0.364).

### 3.6 Clinical outcomes of CNS infections

In Table 5, we evaluated the clinical outcomes of CNS infections between the MHI group and the LI group. The results indicate notable differences in individual outcomes based on immune status. For complete remission, the MHI group demonstrated a significantly higher rate compared to the LI group (*p* = 0.031). In terms of partial remission, there was no significant difference observed between the two groups (*p* = 0.613). However, progression of the CNS opportunistic infection was more frequent in the LI group than in the MHI group (*p* = 0.022).

**Table 5:** Comparison of clinical outcomes between two groups.

Parameter	MHI group (*n* = 101)	LI group (*n* = 112)	χ^2^	*P*
Complete remission [*n*(%)]	67 (66.34%)	58 (51.79%)	4.638	0.031
Partial remission [*n*(%)]	24 (23.76%)	30 (26.79%)	0.257	0.613
Disease progression [*n*(%)]	10 (9.90%)	24 (21.42%)	5.261	0.022

### 3.7 Multivariate analysis

In Table 6, multivariate analysis identified CD4 + T-cell count as an independent predictor of outcome. For every 100 cells/μL increase in CD4 + T-cell count, there is a markedly reduced risk of poor prognosis (OR = 0.321, *p* < 0.001). Conversely, each log10 increase in HIV viral load was associated with an elevated risk (OR = 1.453, *p* = 0.001). The presence of CNS symptoms also indicated a higher risk of poor outcomes (OR = 1.321, *p* = 0.023). Specifically, *Cryptococcus neoformans* infection compared to other pathogens was a significant predictor of worse outcomes (OR = 2.154, *p* < 0.001). Lastly, ART usage at admission appeared protective (OR = 0.563, *p* = 0.026). The model included 7 events per predictor variable, and no significant collinearity was detected.

**Table 6:** Multivariate analysis of poor prognostic factors.

Variable	OR (95% CI)	*P*
CD4 + T-cell count (per 100 cells/μL increase)	0.321 (0.225–0.460)	<0.001
HIV viral load (per 1 log10 copies/mL increase)	1.453 (1.184–1.791)	0.001
CNS symptoms (yes vs. no)	1.321 (1.043–1.682)	0.023
CNS infecting pathogen (*Cryptococcus neoformans* vs. others)	2.154 (1.452–3.214)	<0.001
ART usage rate at admission (yes vs. no)	0.563 (0.342–0.913)	0.026

CD4 + T: Cluster of Differentiation 4-positive T Lymphocytes; CNS: Central Nervous System; ART: Antiretroviral Therapy.

### 3.8 Quality of life scores (QoL)

In Table 7, we compared QoL scores between the MHI group and the LI group at discharge. The analysis revealed QoL scores were higher in the MHI group across all assessed domains. For physical function, the MHI group reported higher scores compared to the LI group (*p* = 0.001). Similarly, psychological function scores were significantly better in the MHI group than in the LI group (*p* = 0.002). Social function also demonstrated a significant difference, with the MHI group scoring higher than the LI group (*p* = 0.002). Lastly, the overall health score was notably higher in the MHI group compared to the LI group (*p* < 0.001).

**Table 7:** Quality of life scores.

Parameter	MHI group (*n* = 101)	LI group (*n* = 112)	t	*P*
Physical function	71.24 ± 12.41	64.75 ± 16.52	3.261	0.001
Psychological function	65.22 ± 14.25	58.68 ± 16.13	3.123	0.002
Social function	62.73 ± 13.53	55.82 ± 18.74	3.105	0.002
Overall health score	63.42 ± 13.20	54.26 ± 17.84	4.283	<0.001

QoL: Quality of life.

## 4 Discussion

This study provides contemporary, detailed quantification of the profound impact that advanced immunosuppression (CD4 < 200 cells/μL) has on the clinical-microbiological profile, treatment response, and quality of life of individuals with AIDS who develop CNS infections, reinforcing and extending the established association between immune status and neurological outcomes. Individuals with CD4 below 200 cells/μL (LI group) exhibited more severe infections, poorer treatment responses, and lower quality of life than those with CD4 at or above 200 cells/μL (MHI group). Our data robustly quantify the steep gradient of risk for severe CNS infection manifestations around the CD4 threshold of 200 cells/μL. While CD4 count is a continuum, these findings underscore the critical importance of this specific cutoff in clinical guidelines for anticipating complications and intensifying diagnostic scrutiny. Future studies with finer immunological stratification could further refine risk prediction within the CD4 ≥ 200 group.

The higher frequency of pathogens like *Cryptococcus neoformans*, MTB, and CMV in the LI group aligns with known immunopathology. CD4 + T-cells, especially the Th1 subset, are important for activating macrophages and other immune cells to fight these infections. Low CD4 counts compromise this response (20). For *Cryptococcus neoformans*, effective clearance needs interferon-gamma (IFN-*γ*) from Th1 cells to boost macrophage activity. Without enough CD4 cells and IFN-γ, fungal control is impaired, which may explain its higher detection rate in LI individuals (21). Similarly, controlling MTB requires CD4 cells to form protective granulomas (22). CMV control depends heavily on CD8 + T-cells, whose function can be impaired in the absence of CD4 help, potentially leading to viral reactivation (11). The lack of difference in JCV likely relates to its distinct pathology (direct oligodendrocyte infection) and different immune control mechanisms (23).

The more abnormal CSF findings in the LI group (high cells, high protein, low glucose) are indicative of stronger inflammatory responses. Low CD4 counts may impair pathogen containment, allowing microbial proliferation and triggering intense inflammation. Immune cell influx into the CNS can release inflammatory mediators and enzymes that may compromise the blood–brain barrier (24). This could lead to increased plasma protein leakage into the CSF, raising protein levels. Inflammation may also disrupt glucose transport into the CSF, lowering glucose levels. The higher white blood cell counts reflect the presence of an immune response within the CNS (25, 26).

Brain imaging abnormalities were significantly more common in the LI group. Brain lesions and enlarged ventricles may indicate tissue injury or atrophy, potentially from direct pathogen damage and inflammation. Meningeal enhancement suggests inflammation of the meninges, commonly seen in infections such as tuberculosis, cryptococcosis, or CMV. Focal lesions like abscesses or tuberculomas may arise when the immune system fails to contain infections locally (27–29). These imaging findings quantitatively demonstrate that the degree of immune compromise, particularly a CD4 count below 200 cells/μL, is strongly associated with a higher burden and variety of structural CNS abnormalities in the setting of active infection.

Consistent with these pathological and inflammatory differences, LI individuals had less favorable clinical outcomes. Their lower rate of complete remission and higher disease progression may be related to uncontrolled infections and greater neurological damage. The multivariate analysis indicated that every 100 cells/μL increase in CD4 count was associated with a lower risk of a poor outcome. High HIV viral load and *Cryptococcus neoformans* infection were also independent predictors of worse prognosis, highlighting their combined association with adverse outcomes in the context of low CD4 counts. Importantly, ART use at admission was associated with better outcomes, emphasizing the potential importance of immune restoration (20, 30).

The lower Quality of Life (QoL) scores in the LI group across all domains (physical, psychological, social, overall health) suggest a tangible impact on individual well-being. Neurological damage can lead to physical disability and cognitive problems, potentially affecting physical and psychological function. Social isolation may occur due to stigma and disability. Persistent symptoms and treatment side effects might further reduce overall well-being. These findings underscore the potential value of preserving immune function for maintaining neurological health and quality of life (31, 32).

Beyond the absolute CD4 count, our data also highlight profound disturbances in broader immune architecture, evidenced by the significantly lower CD4/CD8 ratio in the CD4 < 200 group. While our study design focuses on CD4 count as the primary axis of immunodeficiency, the depressed CD4/CD8 ratio corroborates the state of advanced immune dysregulation in these individuals. Future prospective studies are warranted to evaluate whether the CD4/CD8 ratio, independent of or in combination with the absolute CD4 count, offers additional prognostic value for CNS infection outcomes in the modern ART era.

Our findings should be interpreted within the study’s limits. The retrospective design relied on existing medical records, which may introduce selection and information biases. This is a single-center study in China, which may limit the generalizability of the results to other populations with different healthcare systems, genetic backgrounds, or prevalent pathogens. Furthermore, our dichotomous grouping at 200 cells/μL, while clinically meaningful, is a simplification. The CD4 ≥ 200 group includes individuals with a wide range of counts. Future studies with finer CD4 strata (e.g., 200–350, 350–500, >500 cells/μL) could provide more granular risk stratification. As an exploratory analysis with modest sample size, no adjustment for multiple comparisons was performed and effect sizes were not systematically reported, limiting quantitative assessment of clinical magnitude and increasing false-positive risk. While statistical significance guided initial interpretation, future confirmatory studies should incorporate effect size metrics and rigorous multiple testing corrections to enhance result interpretability and clinical applicability. Although we used standard pathogen testing methods, some infections, especially certain viruses or atypical organisms, might have been missed. Furthermore, detailed antiretroviral therapy (ART) history, including specific regimens, precise duration, and adherence metrics, was not uniformly available or analyzed as a core variable. While we captured ART usage at admission, this heterogeneity in treatment background, reflective of real-world clinical settings, may confound the association between immune status and outcomes. Baseline QoL was not assessed due to the retrospective design. We assessed quality of life only at discharge, longer-term follow-up would be needed to determine if these differences persist. While we adjusted for several key factors in multivariable analysis, other unmeasured variables such as specific ART regimens, drug resistance, psychosocial factors, socioeconomic indicators, or immune reconstitution inflammatory syndrome (IRIS) could influence outcomes and were not captured.

Future research should include prospective longitudinal studies to examine how immune recovery with ART influences long-term CNS outcomes. Exploring specific immune markers beyond CD4 count (like CD4/CD8 ratio, T-cell activation markers, or CSF cytokine levels) could improve risk prediction. Studies comparing the effectiveness of different ART regimens or adjunctive anti-inflammatory therapies for preventing or treating CNS infections are also warranted.

Clinically, our results support several considerations. Early HIV diagnosis and rapid ART initiation remain crucial to preserve immune function. Individuals with low CD4 counts (especially <200 cells/μL) may benefit from vigilant monitoring for subtle CNS symptoms, with a low threshold for CSF analysis and neuroimaging. In high-risk individuals, prophylactic treatments against specific pathogens like Cryptococcus should be considered according to existing guidelines. Finally, management of CNS opportunistic infections should integrate antimicrobial therapy with attention to inflammation control, neurological rehabilitation, and holistic support addressing quality of life (33, 34).

## 5 Conclusion

This study demonstrates that immune status, as measured by CD4 + T-cell counts, is closely associated with CNS infection risk, severity, and outcomes in AIDS individuals. Lower CD4 counts correlate with higher pathogen detection rates, more severe clinical and imaging findings, and reduced quality of life. Future research should focus on elucidating the molecular mechanisms underlying immune-pathogen interactions and developing integrated biomarkers for individual risk prediction. By combining immunological and clinical data, healthcare providers may better stratify individuals and tailor management strategies to improve long-term outcomes in this vulnerable population.

## Data Availability

The raw data supporting the conclusions of this article will be made available by the authors, without undue reservation.
